# Community bylaws concerning sexual and reproductive health and rights in Machinga District, Malawi: to what extent are they responsive to young people’s needs?

**DOI:** 10.1186/s12939-023-02054-7

**Published:** 2023-11-14

**Authors:** Maryse Kok, Alister Munthali, Peter Mvula, Zindaba Chisiza, Marielle Le Mat

**Affiliations:** 1 Department of Global Health, KIT Royal Tropical Institute, Amsterdam, the Netherlands; 2 PALM Consulting Limited, Zomba, Malawi; 3 Department of Drama and Theatre Studies, University of Malawi, Zomba, Malawi

**Keywords:** Sexual and reproductive health and rights, Bylaw, Young people, Malawi

## Abstract

**Background:**

Community bylaws are commonly accepted mechanisms to influence behaviour change to achieve better health and development outcomes in sub-Saharan Africa. However, the uses, benefits, and potential downsides of community bylaws are largely unclear, especially regarding sexual and reproductive health and rights (SRHR) of young people. The objective of this study was to determine the extent to which community bylaws in Machinga District in southern Malawi are responsive to young people’s realities and SRHR needs.

**Methods:**

In Phase 1 of this qualitative study, 35 community members were interviewed, including 14 young people (15–24 years), five parents, five traditional leaders, and eleven key informants. Based on findings from Phase 1, eleven members from local youth groups co-created a drama performance that covered issues concerning bylaws and young people’s SRHR (Phase 2). The drama was performed in the community, after which young women (18–24 years), young men (17–24 years), female and male parents discussed on what they saw in the drama, focusing on young people’s SRHR, in four focus group discussions (Phase 3). All transcripts were coded and thematically analysed and narratives were written on main themes.

**Results:**

Three community SRHR bylaws, related to teenage pregnancy, child marriage, and sexual harassment and rape were identified and commonly accepted in the community. While these bylaws intend to reduce SRHR-related issues among young people, they are often not involved in bylaw formulation. The bylaws were associated with protection of girls, and a good reputation for boys, young men and traditional leaders. Bylaw enforcement faced problems, as fines were not in line with national laws, and wealthy offenders could avoid them through bribes. Effects of bylaws on teenage pregnancy rates seemed limited, while some positive effects on school readmission, prevention of child marriage, and reporting sexual harassment were reported.

**Conclusions:**

The study revealed that community bylaws were accepted but not owned by young people, and had different effects on the rich versus the poor, and girls versus young men. Bylaws were associated with punishment in terms of money, which seemed to overpower their potential to promote rights and address social norms underlying SRHR issues of the youth.

## Introduction

Traditional leadership remains a strong locus of political obligation in sub-Saharan Africa. In Malawi, the traditional chieftaincy, existing alongside the democratically elected local government structures, holds respect and power within communities [1]. Malawi’s traditional leadership has four ranks in the hierarchy: the Paramount Chief, Traditional Authority (TA), Group Village Head (GVH), and Village Head [1]. While traditional leaders have little official power, they play an important role in safeguarding social norms and facilitating community mobilisation. They are often custodians of culture, local customary laws and bylaws [2–4].

Bylaws are commonly accepted mechanisms to influence behaviour change to achieve better health and development outcomes. Bylaws can be defined as “rules and norms to regulate life in communities and to levy fines for the non-compliance to those by-laws” [5]. According to the 1998 Local Government Act of Malawi, the creation of bylaws is under the mandate of the District Council for the “good governance of the whole or any part of the local government area”, and this authority cannot be delegated. Bylaws developed at district level need approval of the Ministry of Local Government [6]. Community bylaws that are created at community level by traditional leaders, often with the support of non-governmental organisations (NGOs), are therefore not officially enforced through the Local Government Act [2, 3]. Nevertheless, there has been an increase in the number of community bylaws in Malawi, partly because international and national laws sometimes fail to result in change at community level as they are not context-specific enough or poorly implemented [3].

Current literature indicates that community bylaws in Malawi focus on combating child labour and promoting natural resources management, education and health. Concerning the latter, various studies mention community bylaws that aim to ensure women attend antenatal care, deliver at a health facility and, in some TAs^1^, go for postnatal check-ups. Several studies in Malawi report on community bylaws aimed at preventing sexual and gender-based violence (SGBV), child marriage, teenage pregnancy, and promoting menstrual hygiene in schools [2–5, 7].

Some of these studies have observed that bylaws indeed contributed to combatting child marriage [2, 4] and increasing reenrolment of girls in school [2]. At the same time, research has documented tensions in relation to bylaws. First, community bylaws sometimes contradict or expand on national laws and policies [2, 8]. Examples include bylaws fining more or less than what is included in national thresholds, and adopting more severe or less strict sanctions than those stated in statutory laws [3]. Second, studies mention a lack of transparency in the use of revenue and inconsistent application of bylaws [4]. Third, bylaws are interpreted and implemented in diverging ways, partly because they are often unwritten and not sufficiently disseminated in the community [2].

Finally, several scholars discuss equity problems of adherence to and punishments of the bylaws. These studies mention that poor people tend to have less means to comply with the rules (such as going to the health facility for delivery) and also have less means to pay potential fines [2, 5, 9, 10]. A study on bylaws that intended to increase male involvement in antenatal care visits, reported that the bylaws in fact additionally burdened women without spouses to seek and pay for support letters from traditional leaders to attend antenatal care alone or with alternative support [11]. In such cases, bylaws can reproduce inequity rather than address challenges of accessing services.

While some positive and negative effects of bylaws have thus been documented in previous studies, the effects of bylaws on health and development outcomes remain largely unclear. In particular, bylaws that relate to young people’s sexual and reproductive health and rights (SRHR) are less studied, including from the perspective of young people themselves. No research has assessed how and to what extent young people are engaged in formulating bylaws that concern them, and to what extent these bylaws respond to young people’s needs. There is also limited evidence on how bylaws might influence social norms on SRHR-related issues concerning young people. It is important to gain insights into these issues, since an increasing number of NGOs and government actors focus on the creation and implementation of bylaws for the promotion of health and development, including young people’s SRHR. Therefore, this study explored the extent to which community bylaws in Machinga District in southern Malawi are responsive to young people’s realities and SRHR needs.

## Methods

This qualitative study took place in one GVH under one TA in Machinga District in southern Malawi. The TA and GVH were selected because the research was commissioned by the Break Free! Consortium that implements an SRHR advocacy programme there, which includes supporting bylaw development and awareness raising on bylaws. Data were collected from September to November 2022 in three phases. In the first phase, 35 interviews with different people were conducted to discuss the bylaw development process, content, interpretation, implementation and effects. Study participants were selected based on their knowledge about what is going on in the community, and therefore, had been living or working in the community. Break Free! staff assisted in identifying appropriate participants. We spoke to:

- Fourteen young people (15–24 years), from different genders, ages, marital status, schooling status and including youth leaders and disabled youth.
- Five parents and caregivers, from different genders and including those involved in initiation ceremonies.
- Five traditional leaders: a TA, a GVH and three village heads.
- Eleven other key informants, including a teacher, two health workers (a medical assistant and a community health worker in Malawi known as health surveillance assistant), one community development assistant, one community child protection worker, a magistrate, a representative from the police, representatives from two NGOs and two staff members from the Machinga District Council.

Data from Phase 1 were coded, analysed, and thematic summaries were developed. These summaries provided input into Phase 2, in which eleven youth group members were recruited, trained and participated in a co-creation of theatre performance. Each young person represented one active youth club based in the study TA. The decision to use drama as method for disseminating our research findings was informed by our desire to enhance community involvement and promote a better social understanding on matters linked to young people, SRHR and community bylaws. Rather than involving an external group of artists to create and present drama plays to the wider community, our approach involved working with active members of youth clubs in Machinga. We trained the youth in theatre techniques, enabling them to create performances focusing the central themes. This approach also helped to capacitate the youth in artistic advocacy, allowing them to utilise the acquired skills to develop future performances addressing issues affecting them, but to also train other youth club members. Moreover, using drama offered an opportunity for both the youth and the communities, who could interrogate the issues further and through performance could examine matters from different angles, facilitating a reflective perspective from which they could gain insights.

**Fig. 1:**
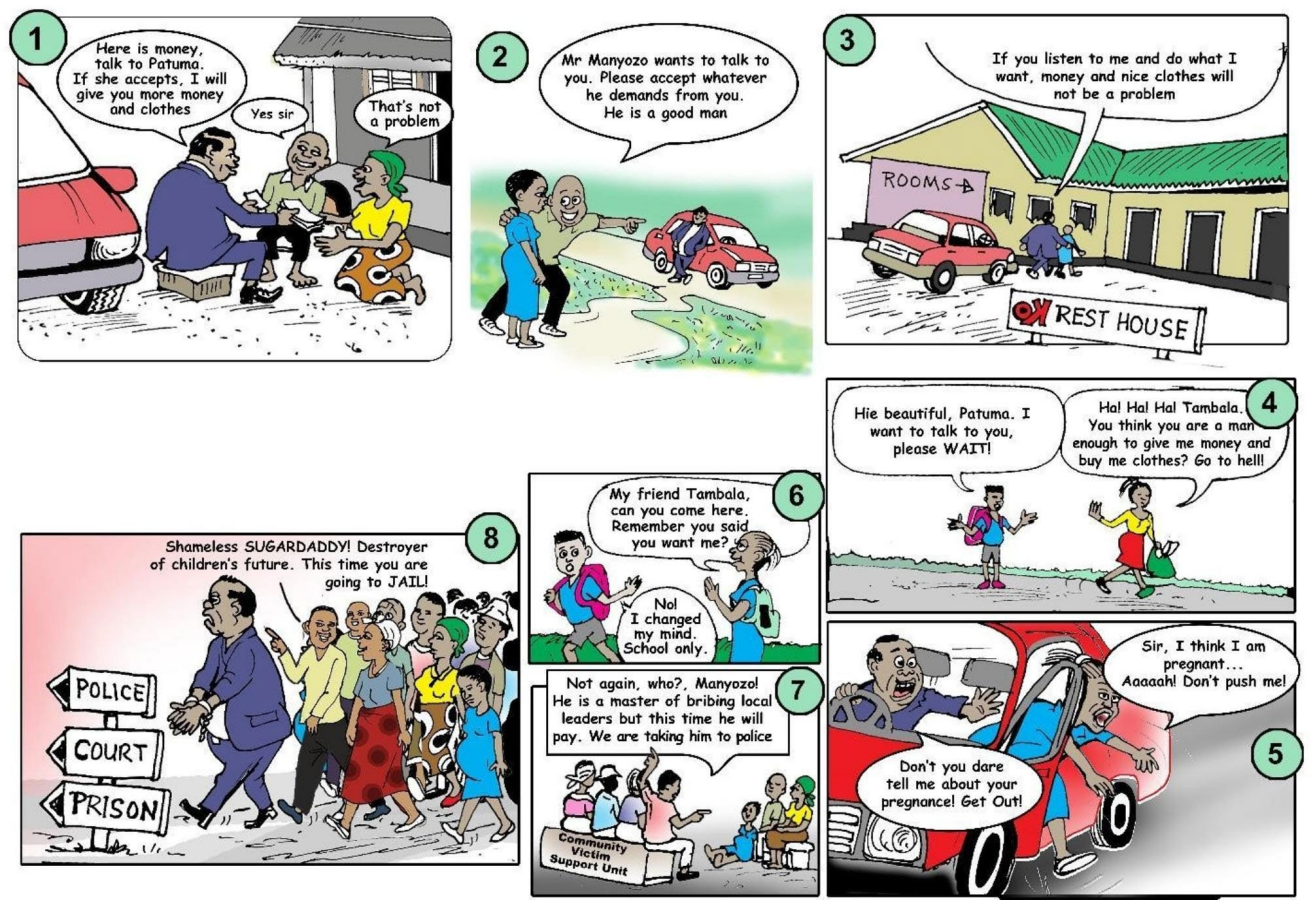
Illustration of the drama main storyline (by James Kazembe)

The youth group members were initially asked to share their lived experiences related to SRHR bylaws. They expressed how the bylaws had impacted them and their peers, using mediums such as songs, dance and sketches. This served to confirm findings from Phase 1 and also allowed those who had not participated in the initial research to contribute their own thoughts on the topic. The group then collaborated to identify the main themes that they wanted to portray through a theatrical performance for the wider community. The group created two plays on bylaws, SRHR and young people. The plays were performed in the local dialect, Yao, which is widely spoken in Machinga. Traditional Yao songs and dance were interwoven into the play, serving to introduce scenes, facilitate transitions and further emphasise the central themes. The play was performed at the headquarters of the TA on 26 November 2022. Figure 1 depicts the main storyline of the drama.

After the drama, Phase 3 of data collection was undertaken: four focus group discussions (FGDs) with young women (18–24 years), young men (17–24 years), female parents/caregivers and male parents/caregivers were conducted, using the same sampling and recruitment strategy as for Phase 1. These FGDs focused on what they saw in the drama play, their opinions, perceptions, and recommendations; with a focus on young people’s SRHR needs.

Data collection tools were pre-tested. Data were collected in Chichewa by a trained research team, which consisted of a mix of younger and older, and female and male researchers. Interviews and FGDs were digitally recorded upon consent of the informants, transcribed and at the same time translated into English. All data were anonymised. Content analysis was carried out using a comprehensive thematic matrix that was developed based on the study objectives and literature review, which facilitated identification of common patterns and trends arising from the narratives. After coding all transcripts in NVivo, narratives were written on main themes.

### Findings

#### The content of SRHR bylaws

Community and key informants mentioned three main SRHR bylaws, which aimed to prevent teenage pregnancy, child marriage, and sexual harassment and rape. The bylaws that aimed to prevent teenage pregnancy and child marriage were interlinked. The bylaws had provisions, rules and fines that applied in cases of teenage pregnancy, child marriage, and sexual harassment and rape. Not all informants had the same account on the rules and fines of these bylaws.

##### What happens when a school-going girl gets pregnant?

Teenage pregnancy, as mentioned by many different informants, is common in Machinga District. A few informants said that if the man responsible for the pregnancy is much older than the girl, he is jailed or fined. If the man responsible is younger, it is more probable that he or his parents will only be asked to provide financial support for the girl and her parents. During an FGD with young men, participants said that boys can also get arrested. A few informants indicated that if the boy is of school-going age, he is also supposed to stop schooling until the girl delivers but this does not always happen. The TA and a village head talked about a related bylaw saying that for school-going girls who are supported by NGOs or the government^2^, if they drop out, for example due to pregnancy, their parents will be asked to refund the goods received and pay a fine of one goat.

##### Preventing and responding to child marriage

Most informants reported that the bylaw concerning child marriage forbids marriage under the age of 18. This bylaw, as stated by informants, aimed at preventing school dropout and severe health consequences of early pregnancy for girls (e.g., fistula). These educational and health-related problems seemed the most prominent justifications for disallowing child marriage and teenage pregnancy. A few informants reported on primary preventative provisions in the bylaw i.e., that pastors and sheikhs who officiate marriages are obliged to verify the age of the girl before officiation and report to the village head. Village heads should keep track of all marriages in their community and ensure that none of them involve children under the age of 18.

“Yes, there are bylaws that say that if a girl wants to get married, she needs to seek approval from the village head who is to confirm her age. The chief and girl have to sign in a book to confirm that indeed the girl is eligible for marriage”, (36-year-old male village head).

If people hear about a planned marriage involving a minor, this could be prevented by talking to the parents and couple involved. The bylaw included rules and fines that mainly seemed to target the parents of the girl (Table 1). Actors involved in trying to resolve or reinforce these rules and fines were traditional leaders, the community victim support unit (CVSU) and NGOs. CVSUs are structures established at TA level, which provide gender-based violence services at the community level and act as a safe haven for victims of violence and abuse. These units also ensure the rehabilitation of victims, especially focusing on women and children.

**Table 1:** Fines in case of child marriage

Parents of the girl	Pastor/ Sheikh	Village head
• To pay 2 goats (MK 40,000) to the Chiefs Forum or community victim support unit (CVSU), or • Disowned from cultivation land, or • Brought to CVSU and then to police to get arrested	• Taken to police or Chiefs Forum/ Council • To pay 2 goats (MK 40,000) • Forced to stop their spiritual activities	• To pay 2 goats (MK 40,000) to the Chiefs Forum

##### What should happen in case of sexual harassment or rape?

Many informants reported that cases of sexual harassment and rape were reported to the CVSU who take them to the police, hospital and court for official handling. These are not handled by traditional leaders.

### Formulation of SRHR bylaws

Most informants reported that the bylaws are written and kept by the chiefs, but are not very detailed. One key informant explained that many community members are illiterate, and are, therefore, verbally informed about the bylaws. The process of formulating the bylaws started with NGOs that visited the TA, who discussed challenges in the community (e.g., teenage pregnancy, child marriage, and school dropout) and explained the need to formulate bylaws to address these challenges. The TA then called for a meeting with GVHs, village heads and other participants such as NGOs, police, the CVSU, the area development committee (ADC), village development committees (VDCs), youth, mother groups, church leaders, health workers, community child protection workers, teachers and other community members.

“Indeed, we have bylaws in this community. The bylaws we have in this community were formulated by us. It was not like we invited somebody from Blantyre to formulate the bylaws for us. We did it on our own”, (GVH).

While some informants stated that youth participated in the formulation of bylaws, others specified that their participation was actually limited: they were invited to a meeting and were just informed about the bylaws.

“He [the chief] invited both boys and girls, and then began telling us that there are bylaws, like a child should not get married when not of age, he/she should be going to school, parents need to encourage their children to be going to school…, that’s it”, (16-year-old in-school boy).

A few informants thought that government officials, together with the chiefs, formulated the bylaws. Subsequently, the chiefs called for village meetings where community members were informed about the bylaws, including the associated fines or punishments for offenders.

“… Then we called for a village meeting whereby we then informed the members of the community of the bylaws that we had come up with. We informed them about bylaws to do with school, child marriages and the like”, (Village head).

### Awareness and interpretation of SRHR bylaws

While most informants were aware of the existence of the bylaws, many of them did not know their exact content. Almost all informants agreed that the bylaws are welcomed and accepted by all community members regardless of age, gender, or status. However, for most informants there was no sense of ownership of the bylaws. That is, they did not feel actively involved in the formulation or application of the bylaws in their community. Most informants including a GVH interpreted the purpose of the bylaws to be protecting the lives of young people, particularly of girls and young women.

“I think it’s for protection (…); If a girl becomes pregnant at a tender age, it means we have killed that girl. Once a girl has a child, poverty sets in. She cannot do anything. It means she has to support the child born to her. Since she is not mature enough to have a baby, they end up having complications and others also die. This is the reason why we came up with this rule”, (22-year-old unmarried man).

While some young women in the FGD emphasised that they feel protected as a result of the bylaws, young men emphasised fear for their reputation if they failed to adhere to the bylaws. Indeed, some considered that bylaws serve to instil fear in young people, parents, and other key actors to prevent child marriage, teenage pregnancy, and sexual violence from happening. One informant stated that those who have trespassed are seen as morally inferior in the community.

“To me, [the bylaws] are good. (…). The coming in of this bylaw, it saves girls in the community. If a man has raped a girl, it means the man has no moral values. He has done contrary to the bylaw”, (Ngaliba^3^).

Many informants were of the view that young people needed to be protected through these bylaws, so that they can be healthy and educated citizens who can effectively contribute to development.

“… to finish our education. If we make it, then we will be able to develop this community or even the whole Malawi“, (17-year-old in-school boy).

The fact that community development was a central concern was also reflected in the response of a GVH who took the level of development gains as a reflection on his performance.

“…. the laws are helping me with my chieftaincy and good reputation. If a community is failing, the one being pointed at is the Group Village Head for the community for the failure.” (GVH).

This GVH went on to thank the Break Free! programme for the support by initiating the bylaw formulation in the community.

### Implementation, adherence, and enforcement of SRHR bylaws

A wide range of stakeholders were involved in the implementation and enforcement of bylaws and these included the TA, GVHs, village heads, Chiefs’ Council, child protection worker, CVSU, community police, police, NGOs, mother groups, health workers, youth, parents, and other community members. The roles of these people and institutions are described in Table 2.

**Table 2:** Roles of various stakeholders in implementation and enforcement of bylaws

Stakeholders	Roles
TA, GVHs, village heads	• Key in the enforcement of bylaws. ○ Remind community members about the bylaws, especially during village meetings. ○ Handle cases and refer them to relevant authorities including the police, child protection worker or CVSU.
Chiefs’ Council	• Helps to see that the bylaws are being adhered to.
Child protection worker	• Creates awareness about bylaws. • Attends Chief’s Council and is the one who deals with children’s issues in the Council. • Ensures that children are protected, in terms of abuse, child marriage and avoiding school dropout.
CVSU	• Manages cases of child abuse; hence helping with enforcement of bylaws.
Community police	• Monitors compliance with the bylaws in the communities. • Takes cases to the police.
Police	• Helps to enforce the bylaws i.e. ensuring that the bylaws are adhered to by community members. • Arrests and jails offenders.
NGOs	• Sensitise community members about the bylaws. • Encourage community members to adhere to bylaws.
Mother groups	• Follow up cases of child marriage and school dropout and discuss these with parents. • Mobilise resources e.g., buying notebooks for school-going children.
Health workers	• Test girls withdrawn from marriage for sexually transmitted infections or pregnancy. • Encourage youth to go to health facilities, especially for contraceptives.
Youth	• Create awareness about bylaws through drama. • Advise each other and sometimes adults on the need to adhere to bylaws. • Go around the village and search for/ investigate cases where bylaws have been broken and report this to the village head.
Parents	• Encourage their children and other community members to adhere to the bylaws. • Report the cases where their children have been abused to authorities including the police; and report cases where bylaws have been broken to the village head.
Community members	• Adhere to community bylaws. • Report cases where bylaws have been broken to the village head and other structures.

Many informants were of the view that the onus of enforcing the bylaws lies with the traditional leaders: village heads, GVHs and TA; and that these bylaws are being implemented and are being adhered to. There were also some community members who did not adhere to the bylaws for several reasons. During the FGD with female parents, a participant reported that some parents do not respect village heads, in some instances parents threaten that they will bewitch the village head.

“They [parents] can seek charms just to deal away with you because you are disturbing their children, because the village head is forcing their children to continue with school. They can even ask the village head like ‘did you yourself go to school?’ Now if my child is able to read that’s enough”, (FGD with female parents).

The bylaw on child marriage was mentioned as not being adhered to for reasons related to the desire for parents to have grandchildren as well as for financial reasons.

“At 18 years a boy marries while for a girl, it is not so. Though we have these rules, for the girl child it is not applied. Mothers once they prefer to have a grandchild, they go for it. I have seen mothers forcing their daughters into marriage so that they should have grandchildren. They force them too much to the point of NGOs giving up. The end result, the NGOs down tools and she marries”, (22-year-old unmarried woman).

This was also stressed during the FGDs after watching the drama performance: participants argued that parents admire their friends who have grandchildren; so, they need to have grandchildren as well. Poverty was another issue that drives child marriage. During the FGDs, participants reported that it is not good for parents to arrange marriages of their children in order for them to financially benefit.

“Patuma’s [referring to a fictional girl in the drama play] parents were interested in receiving money from Mr. Manyozo [fictional man in drama play], forgetting that receiving the money would put their daughter, Patuma, at risk [of engaging in sex with Mr. Manyozo, getting pregnant and dropping out of school]”, (FGD with male parents)

FGD participants acknowledged that they know about girls aged 11 or 12 who are or are forced by their parents into sexual relationships or marriage with someone working in South Africa, because they want to financially benefit from such relationships.

“I know of a case whereby a man who is in Johannesburg, South Africa, wanted a young girl to marry. A certain young girl was in school and her parents forced her to drop out of school and accept the marriage proposal of the man from Johannesburg. This girl did as her parents advised her. Then the man processed her travel documents and then sent her transport money to join him in Johannesburg. Currently, the girl is now in Johannesburg with the man”, (FGD with young women).

The other challenge with adherence to bylaws on child marriage was that some parents lie about the correct age of their children. For instance, in order to avoid being fined by the bylaw, parents would state their child’s age as higher than it actually is. One participant stated:

“They [parents] lie that ‘my daughter is 18 years old’ and yet in real sense she is not. They just want to be favoured and give way for their daughter to get married.“ (Young man).

In the FGD with male parents, participants also discussed a case in which a boy made a girl pregnant, both got fined according to the bylaw, but the boy and girl ended up getting married after all. The other challenge, as mentioned by an NGO worker, a police officer and a village head, was that the bylaws are yet to be endorsed by the District Council and hence difficult to implement. For example, the fines as stated in the bylaws are often not in line with the Constitution and other laws, which hinders effective bylaw enforcement. Nevertheless, bylaws seem to be implemented, despite the inconsistency in fines between national law and community bylaw, although not all community members could afford the fines.

“And even these bylaws can be challenged in the court of law, because they can give you a fine which is above the fine of the same case within our legal system”, (NGO representative).

The TA indicated the same and shared that therefore, the District Council told him that some cases should be left to the police to handle using their legislation. A district officer thought that bylaws are there because people have little knowledge and understanding of national laws.

“… the available laws by the government, most people on the ground do not really understand them. These people are rarely reached with the laws, and even the people that are supposed to take the laws to the people do not understand them, so that’s a complication. Now in the community when they formulate their bylaws, they are like regulating them, so it’s like they are dealing with that problem of not being reached with the government laws… So, if anything, there has to be a good procedure of taking these government laws that are in the Constitution to the people. There should be good procedures for them to understand, only then can we decide to drop the bylaws… The bylaws are coming in because the government laws are not trickling down well to the communities”, (District officer).

Indeed, an NGO representative indicated that the Ministry of Gender, Community Development and Social Welfare developed a booklet on Consolidated Child Related Laws, and that instead of promoting bylaws, this booklet should be the basis for communities’ rules. He recognized that bylaws are good as they cement the already present legal systems in Malawi, but that discrepancies should be taken out, emphasising that bylaws should be re-aligned with the national laws.

A few informants reported other challenges in the implementation of bylaws, such as the lack of transport for child protection workers and youth champions who visit different communities and report cases to the chiefs. Informants also mentioned the existence of bribes paid to those in authority by perpetrators of offences in order to avoid punishment. They reported that the amount of money paid to authorities ranges from MK50,000 to MK100,000. In such cases, the bylaws reinforced existing power relations since those who afford can bribe and thus can ‘risk’ ignoring or violating the bylaws.

“Yes, these things happen. We have a certain village whereby a teacher raped a girl. The teacher went to the chiefs and rulers bribing them so that the case should not be heard. Nevertheless, he failed because the case was reported to the police. Police came to his house to arrest him; he was nowhere to be found. He ran away”, (FGD with young men).

During the drama play, FGD participants observed that an older man proposed to a school-going girl, made her pregnant and when cornered, he wanted to bribe authorities so that he would not face the law.

“Mr. Manyozo was boasting that he knows Patuma together with her parents, because he has been feeding the whole family. He was not fearing going to the court or being arrested because he had a lot of money that would give him freedom to do whatever he wanted to do”, (FGD with male parents).

Many FGD participants shared that such scenarios where community leaders, who are supposed to be trusted, are bribed by offenders indeed happen in their communities. One young man reported that he has been taking economic and social responsibility for a girl he made pregnant. He classified it as a form of corruption, because he escaped the consequences of the bylaw (fines) by arranging with the girl and her family that he would support her (who, as a result, did not press charges against him). This illustrates the inherent conflict of trying to address social norms by instilling bylaws that monetize disobedience to the desired social norms.

### Use of revenue from fines paid after breaching the bylaws

Informants had varying ideas about what is done with the fines paid by offenders. Most informants reported that fines are paid to the chief or the CVSU. A few informants stated the fines will go to the victim, either through the CVSU or directly, and others stated that the chiefs use it for community development. According to the GVH, the money is not used by individuals or the chief, but it is used for community work including helping the needy. There were some informants who said that the money that is paid is shared between the CVSU and the victim.

“Okay, the money is shared among the officers. If it is MK100,000 then they share among themselves MK60,000 and then the MK40,000 remaining, they give it to the parent’s daughter. They take some amount and share among themselves”, (FGD with young men).

Finally, some informants did not really know what the fines were used for.

### Effects of SRHR bylaws on young people and communities

Some members of the community reported that, because of the implementation of the bylaws, fewer girls get pregnant and more girls return to school after delivering.

“…. when a girl gets pregnant, she does not get married but she is told that there is no marriage and that she should wait until she delivers and then she can get back to school after that”, (FGD with male parents).

Some informants reported that generally the number of school-going children has increased: during the FGD with female parents, participants emphasised that because of the bylaws, the number of girls in school has increased from three to seven out of ten. A ngaliba, for example, reported that it was hard for learners in Machinga to write the final primary school examinations, but with the coming of the bylaws, there are now some who are sitting for these exams and that there are now more young people going to secondary school. During the FGD with male parents however, some participants said that in their area not many girls who dropped out of school due to pregnancy returned to school after delivery. They recalled two such cases, arguing that it is difficult to force a girl who has delivered to go back to school. Despite several informants reporting that access to contraceptives has improved, they also reported that nothing has changed regarding teenage pregnancy. Many informants, including a village head and a 40-year-old parent, reported that age at marriage has changed due to the coming of NGOs and that many child marriages have been annulled since the establishment of the bylaws.

“What has changed is that when children get married, the CVSU is able to visit them and annul their marriage, which never used to be the case in the past”, (FGD with adult men).

One other reported effect of the bylaws was that cases of SGBV, including rape, are reported more often.

“Young people were raped and the issue was never dealt with. But currently, rape cases are not kept as a secret and are pursued further”, (VDC member).

Some informants reported that cases of rape or touching of body parts of women are going down e.g., as narrated during the FGD with young women, who further explained that this is because men are afraid of being arrested.

One thing that has changed due to the bylaws is that school and religious books are now being provided in the initiation camps which was not the case previously.

“… The camps we are having now, they are different. We give them books to read. We do not encourage them to go out there and have sex after being initiated. We do ask them which class they are. We give them books to read according to their classes. ‘Which class are you?’ ‘Standard 4’ and then we give them standard 4 books to study”, (Ngaliba).

Previously, initiation ceremonies coincided with the school calendar, which resulted in some learners missing classes. A district officer reported that this is no longer the case because of the bylaws in place. Young men and women in the FGDs as well as the GVH also said that sex after initiation (kusasa fumbi) is no longer promoted.

“What was practised before in terms of cultural practices has changed a lot. Before the laws, children were encouraged to have sexual relations, but now things have changed”, (GVH).

Two young men (20 and 22 years old), however, were critical and said that adolescent boys and girls at traditional initiation camps are still told to experiment with sex. A ngaliba confirmed that in traditional camps in the bush, adolescents are indeed told to experiment with sex. He himself was only involved in modern boys’ circumcision at the hospital, where advice to experiment with sex is not provided anymore. During the FGD with young women, participants also reported that the practice of chimwamazira, in which men would be having sex with their [step] daughters, has also ended with the coming in of NGOs and the bylaws.

One village head was of the opinion that democracy destroyed social norms. He implied that years back, child marriage was uncommon, but that with the coming of democracy, freedom led to people doing what they desired, including having sex and marrying at a young age. Another 36-year-old male village head also said that the community social norms do actually not allow child marriage, but that the problem is that young people do not abstain from sex, and that is why they are getting pregnant and marry early. This implies that the existing norm is that when people have a relationship or are pregnant, then they need to marry – and that the bylaw goes against this social norm.

As much as these changes in norms and cultural practices are encouraging, the data reveal that the bylaws seem to have had little effect on addressing social norms related to teenage pregnancy and child marriage. Even though community informants reported a reduction in child marriage, it is questionable to what extent social norms underlying the practice have changed, because people were reported to lie about their children’s ages and were finding creative ways (including bribes) to proceed with child marriage. Avoiding child marriage, or conducting them in a hidden way, seem to be a result of fear for the bylaw consequences, more than a change of social norms. Particularly, the social norm of having to marry when being pregnant is still supported by many.

While girls can be withdrawn from child marriage, there are unintended effects: a health worker explained that when a girl marries because of poverty and is withdrawn from the marriage, she can fall back into greater poverty. In principle, she is sent back to school, but there are no school fees. He argued that this challenge needs to be addressed. A key informant reported that, with the bylaws, youth are forced to use contraceptive methods to avoid unintended pregnancies, because they fear that if they are found pregnant, “they will face punishment”. However, contraceptive use seemed not easy for all youth. For example, one 17-year-old male informant who was in school said that while young people have access to sexual and reproductive health services, there are challenges regarding actual use of contraceptives.

“They [girls] do not want to use any of the contraceptives, because they say they want to know if they can become pregnant or not. Once they use the contraceptives, they will never know if they are fertile or not”, (17-year-old boy).

This quote shows that misconceptions about contraceptives are still present among the youth, and can make contraceptive use, and thus preventing teenage pregnancy, challenging. It is questionable to what extent a bylaw can address this.

## Discussion

This study revealed three community SRHR bylaws in Machinga District, related to teenage pregnancy, child marriage, and sexual harassment and rape. These bylaws were commonly accepted in the community, but people’s ownership was limited and there were differences in interpretation of the bylaws. While the bylaws intend to reduce SRHR-related issues among young people, they were often not involved in their formulation and implementation – it was mostly driven by traditional leaders, NGOs and adults. The study revealed mixed adherence to the bylaws. For example, prevailing social norms around the importance of marriage and having (grand)children and poverty led some community members to marry off their daughters young. The bylaws were associated with the protection of girls, and a good reputation for boys, young men and traditional leaders. When bylaws were enforced, offenders were punished with fines, often in the form of paying with chickens or goats. However, enforcement also faced problems. The community bylaws were not endorsed by the District Council as required by the Local Government Act and fines were not in line with the national law, which has been discussed by others in Malawi [2]. Therefore, the police and other government officials could not support the local rulings and fines. Moreover, wealthy offenders could avoid fines through bribing the traditional leaders or parents of the girl involved. Revenues of the fines mostly went to the leaders or people involved in CVSUs, and were reportedly only in a few cases used for the benefit of the youth or community at large. Effects of bylaws on teenage pregnancy rates seemed limited, while some positive effects on school readmission, preventing of child marriage and reporting sexual harassment and rape were reported, as confirmed by a few other studies [2, 4].

Other studies also found that bylaws are generally accepted by community members in Malawi [2, 3, 10]. Besides the recognition among community members that bylaws aim to prevent sexual and reproductive health-related issues among young people, Walsh (2018) argues that the acceptance of bylaws is also out of respect for the traditional leadership and in line with Malawi’s hierarchical society [10]. Historically, traditional leadership has been dispensing justice based on unwritten customary law to settle quarrels and punish the guilty in the community [12–14]. Furthermore, payments to traditional leaders (not related to punishment) is common in Malawi, for example in relation to initiation ceremonies, marriage, and first menstruation [15, 16]. While study informants discussed some positive effects of the bylaws on the SRHR for young people, this study revealed that several aspects around regulations and implementation of the bylaws were not responsive to young people’s realities and SRHR needs.

Young people’s voice in the formulation but also in the implementation of community bylaws was largely neglected, which contributed to different interpretations regarding what should happen to offenders, and to little ownership of the bylaws. It also implies that existing power relationships, where adults have more voice than the youth [17], are largely upheld in the bylaw formulation process. The three SRHR-related bylaws were believed to protect girls, which was positively assessed by the informants. However, the bylaws have a different association for boys and young men, because they are almost automatically presented as potential offenders who risk being fined. Adhering to the bylaws is a matter of reputation for them. While this might prevent offences such as sexual harassment, the profound focus on girls’ protection and boys’ reputation does not fully reflect young people’s broader SRHR, and it reflects existing gender inequalities [18]. One key informant spoke about the potential negative effects of child marriage nullification for girls from very poor families. This study reveals that there was a lack of follow-up on what happens to girls after bylaws were applied. Furthermore, results indicate an inherent conflict of trying to address certain social norms by instilling bylaws that monetize disobedience to the desired social norms. That is, bylaws as they are implemented now do not seem to deal with the root causes of inequalities, which ultimately affect the SRHR of young people.

Many studies from Malawi report about maternal health-related bylaws having negative effects on health seeking behaviour, because of the obligation of bringing an (paid) authorization letter from the chief when visiting antenatal clinics without a husband [2, 3, 5, 11, 19, 20]. While this was not the focus of the current study, teenage pregnancy could result in such a situation. In an earlier qualitative study in Machinga District, a few key informants reported that unsafe abortion went up because of fear for the bylaw fines [21], however, this was not found in the current study.

The issue of bribing duty bearers has also been reported in other studies [4, 22]. Incorrect enforcement of bylaws aggravates the difference between the rich and the poor in the community, as the rich can stay unpunished, while the poor do get punished but have less means to pay the fines [2, 5, 9, 10]. In addition, the findings show that relatively wealthier and more powerful people financially benefit from the bylaw fines, more than poorer and less powerful people – including the youth. There seems to be less focus on what happens to victims or young people involved in the cases being judged upon, and more focus on the money. Another study from Malawi confirms this: people with a certain position in the community, such as health workers, issued fines in the name of the bylaw, while it was not part of the bylaw, to make personal financial gains [5].

The above shows that bylaws in Machinga District have not (yet) resulted in substantial behaviour change. Underlying social norms concerning the voice of the youth, marriage, reproduction and gender are not easy to change. Traditional leaders’ roles as custodians of social norms and cultural practices are, according to some, in contradiction with their roles in pioneering community bylaws that oppose harmful traditions and practices and promote human rights [3], especially if such bylaws are initiated from outside. Bylaws are not a magic bullet to address community problems such as teenage pregnancy, child marriage and school dropout. NGOs, traditional leaders and other stakeholders should combine the implementation of bylaws with other interventions aiming at social norm change and economic empowerment. Further, all stakeholders involved should have more eye for the rights perspective when facilitating the establishment and implementation of bylaws, to avoid bylaws reinforcing existing inequalities.

The subject of community bylaws is contextual, therefore, the study findings are not directly applicable to other communities in Malawi and beyond, yet similarities with other contexts have been identified and discussed based on the literature. Informants were generally willing to share their views, however, some realities might have been missed because of socially desirable answers around sensitive topics. The strengths of this study are the involvement of a wide variety of informants and the participation of young people in creating one of the study methods (the drama).

## Conclusion

Bylaws in Machinga District, southern Malawi, were associated with punishment in terms of money, which seemed to overpower their potential to promote equality, rights and address social norms underlying SRHR issues of the youth. As bylaws are established and accepted in the community, it is important to improve them so that they are in line with national laws, better enforced and more focused on addressing young people’s realities and SRHR needs. This requires the involvement of traditional leadership, NGOs, the Government and the community, and, especially, the youth.

## Data Availability

The dataset used and analysed during the current study are available from the corresponding author on reasonable request.

## Footnotes

1 TA refers to both the area and the TA rank in traditional leadership.

2 Some NGOs support girls to go and/or stay in school through providing educational materials including uniforms, notebooks, pens and school bags.

3 A ngaliba is a man who circumcises the boys.

## Abbreviations

ADC: Area Development Committee
CVSU: Community Victim Support Unit
FGD: Focus Group Discussion
GVH: Group Village Head
NGO: Non-Governmental Organisation
SGBV: Sexual and Gender-Based Violence
SRHR: Sexual and Reproductive Health and Rights
TA: Traditional Authority
VDC: Village Development Committee
